# Moderate AMPA receptor clustering on the nanoscale can efficiently potentiate synaptic current

**DOI:** 10.1098/rstb.2013.0167

**Published:** 2014-01-05

**Authors:** Leonid P. Savtchenko, Dmitri A. Rusakov

**Affiliations:** UCL Institute of Neurology, University College London, Queen Square, London WC1N 3BG, UK

**Keywords:** AMPA receptor, NMDA receptor, long-term potentiation

## Abstract

The prevailing view at present is that postsynaptic expression of the classical NMDA receptor-dependent long-term potentiation relies on an increase in the numbers of local AMPA receptors (AMPARs). This is thought to parallel an expansion of postsynaptic cell specializations, for instance dendritic spine heads, which accommodate synaptic receptor proteins. However, glutamate released into the synaptic cleft can normally activate only a hotspot of low-affinity AMPARs that occur in the vicinity of the release site. How the enlargement of the AMPAR pool is causally related to the potentiated AMPAR current remains therefore poorly understood. To understand possible scenarios of postsynaptic potentiation, here we explore a detailed Monte Carlo model of the typical small excitatory synapse. Simulations suggest that approximately 50% increase in the synaptic AMPAR current could be provided by expanding the existing AMPAR pool at the expense of 100–200% new AMPARs added at the same packing density. Alternatively, reducing the inter-receptor distances by only 30–35% could achieve a similar level of current potentiation without any changes in the receptor numbers. The NMDA receptor current also appears sensitive to the NMDA receptor crowding. Our observations provide a quantitative framework for understanding the ‘resource-efficient’ ways to enact use-dependent changes in the architecture of central synapses.

## 1. Introduction

Cellular mechanisms of use-dependent synaptic plasticity remain a subject of intense investigation, simply because they hold a promise to unveil the neurobiological basis of learning and memory formation in the brain. The molecular machinery underlying the classical plasticity paradigm, long-term potentiation (LTP) of AMPA receptor (AMPAR)-mediated synaptic transmission, has been at the centre stage of neuroscience research for decades [1–3]. The currently prevailing view is that the expression of classical NMDA receptor (NMDAR)-dependent LTP relies on the increased postsynaptic AMPAR current, although a boost in presynaptic release probability has been found important at least in some physiological scenarios [4–6]. The mechanism providing the LTP-associated AMPAR current increase is thought to involve activity-induced insertion of additional synaptic AMPARs through either endocytosis or lateral membrane trafficking, or both [7–12]. This paradigm could, in principle, explain a variety of physiological phenomena associated with a use-dependent functional and structural plasticity of excitatory synaptic connections [13].

At the same time, various theoretical models of small excitatory synapses seem to converge on a prediction that glutamate released into the synaptic cleft from a synaptic vesicle activates in most cases only a relatively small hotspot of local AMPARs [14–19]. Consequently, it has long been understood that the pattern of AMPARs within the synaptic cleft could significantly affect the synaptic current amplitude. However, whether morphologically plausible changes in the receptor distribution inside the cleft could actually produce synaptic potentiation consistent with the experimental LTP has remained unclear. Similarly, the question arises whether the insertion of additional AMPARs simply by expanding the postsynaptic density (PSD), as some LTP scenarios appear to suggest, is a naturally efficient way to increase synaptic strength.

Obtaining reliable experimental evidence for an LTP-associated increase in the local AMPAR numbers or their density on the nanoscale has not yet been technically feasible. Where, within the PSD, and how many additional AMPARs are normally required to explain robust synaptic potentiation remains therefore poorly understood. To address these questions in a quantitative manner, here we explore the relationship between the arrangement of AMPARs (and NMDARs) and synaptic efficacy at common excitatory synapses (exemplified by the CA3–CA1 connection in the hippocampus) using a computational Monte Carlo model. We adapt the modelling approach which has been tested and validated extensively against the experimental data including sub-millisecond probing of native AMPARs and NMDARs in outside-out, somatic and dendritic, membrane patches [18,20–23]. In our simulations, we systematically explore changes in the density and numbers of synaptic AMPARs (and NMDARs) and examine the ensuing effect on the amplitude of synaptic currents. We have to stress here that this study focuses on a widely held, archetypal LTP paradigm which is confined to an individual synapse and has been routinely explored in various experimental circumstances, from electron microscopy to dendritic spine imaging studies. The LTP mechanisms which involve changes in the composition of synaptic population, for instance conversion of silent synapses, are outside the scope of the present analysis. In assessing the reliability of our theoretical predictions, we routinely test whether such predictions remain robust over a physiological range of synaptic parameters that are difficult to access empirically.

## 2. Material and methods

### (a) Monte Carlo model: synaptic architecture and environment

**Figure 1.**
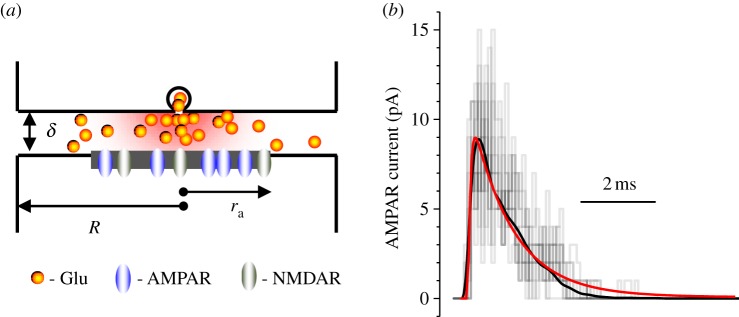
Simulations of glutamate release, diffusion and receptor activation in the environment of small central synapses. (*a*) Model schematic; see §2*a* for notation and [20,22] for algorithm details and experimental validation. (*b*) An example of AMPAR current simulations in response to release of 2700 glutamate molecules (postsynaptic membrane potential *V*_m_ = −80 mV). Grey staggered traces, 10 example simulation runs depicting stochastic activation of individual AMPARs by individual glutamate molecules, as further detailed in [18]; black trace, an average trace of 40 simulation runs, with stochastic receptor activation; red trace, simulation outcome with AMPARs activated by the average glutamate concentration calculated from the number of glutamate molecules in the vicinity (to reduce computation time).

We adapted the modelling methods which have been tested and validated previously with respect to the architecture and physiology of CA3–CA1 synapses [18,20,22,24]. Computations were carried out using an in-house 64-node PC cluster optimized for parallel computing [18] (algorithms provided by Sitrus LLC, Boston, MA, USA). In the model, the synaptic apposition between adjacent pre- and postsynaptic membranes was represented by a flat cylindrical cleft with radius *R* ranging from 180 to 240 nm (figure 1*a*). This included the PSD (the membrane area which contains randomly scattered synaptic AMPARs and/or NMDARs and is opposed by the presynaptic active zone where release occurs) with radius *r*_a_ ranging from 60 to 200 nm (figure 1*a*). The diffusion coefficient of glutamate inside the cleft was set at 0.33 μm^2^ ms^−1^, and 0.4 µm^2^ ms^−1^ outside the cleft, in accord with previously published estimates [18,22,25]. The synaptic cleft height *δ* = 19 nm was set to reflect the average distance between the electron density maxima of the pre- and postsynaptic membranes in electron micrographs of CA1 synapses [20,26]. Within the postsynaptic active zone, the total number *N* of AMPARs (channel conductance 12.5 pS) or NMDARs (25 pS) varied between 15 and 110 or 2 and 30, respectively, consistent with quantitative immuno-electron microscopy [27,28] and with the range estimated by optical quantal analyses [29] at these synapses. Brownian movements of individual glutamate molecules and receptor state transitions following activation were computed with a time step of 0.1 μs; further reduction of the time step by an order of magnitude improved computation accuracy by less than 1%; all kinetic receptor states were recorded and stored.

### (b) Monte Carlo model: release of glutamate

A total of 2700 glutamate molecules were released into the cleft quasi-instantaneously, according to the value estimated in a recent experimental study of CA3–CA1 synapses [22]. In most cases, glutamate was released at the cleft centre, in accord with a traditional modelling approach that routinely accounts for the problem's rotational symmetry. However, we also explored the scenario in which the release site was distributed randomly within the active zone. In these latter simulations, the average amplitude of the AMPAR or NMDAR currents (and its standard deviation) was calculated for 20 consecutive simulated release events, as indicated.

### (c) Monte Carlo model: receptor activation

The working algorithm, which has been detailed earlier [22,24,30], calculates receptor kinetics from average concentrations of local glutamate represented by individually tracked molecules in the vicinity of the receptor. This hybrid algorithm requires much less computational resources compared with the Monte-Carlo-throughout method which computes explicit stochastic behaviour of individual receptors activated by individual glutamate molecules [18] while giving a virtually identical average outcome (figure 1*b*). In brief, at each time step (d*t* = 0.1 µs), the model updated the coordinates of all individual glutamate molecules that follow Brownian movement. Next, it calculated the concentration profile of glutamate *C*(*r*,*t*) in the cleft. In conditions of approximate rotational symmetry, this corresponded to *C*(*r*,*t*) = *N*_*δ*_(2**π*r*δ*Δr*)^−1^, where *N*_δ_ stands for the number of glutamate molecules occurring at time point *t* inside the flat cylindrical ring of height **δ**, width *Δr* and radius *r*. The average occurrence (concentration) of open receptors [*O*](*r*) within the active zone (*r*<*r*_a_) was then calculated at the same time point from the multi-stage AMPAR kinetics [31] or NMDAR kinetics [32] adjusted for 33–35°C using the immediate history of the receptor states and *C*(*r*,*t*). These calculations gave the total synaptic current (*V*_m_ = −80 mV for AMPARs, no Mg^2+^ block for NMDARs) integrated over the PSD area. The duty cycle was repeated systematically throughout the model run.

## 3. Results

### (a) Potentiation requires a disproportionately large number of AMPARs added to the PSD at the same density

**Figure 2.**
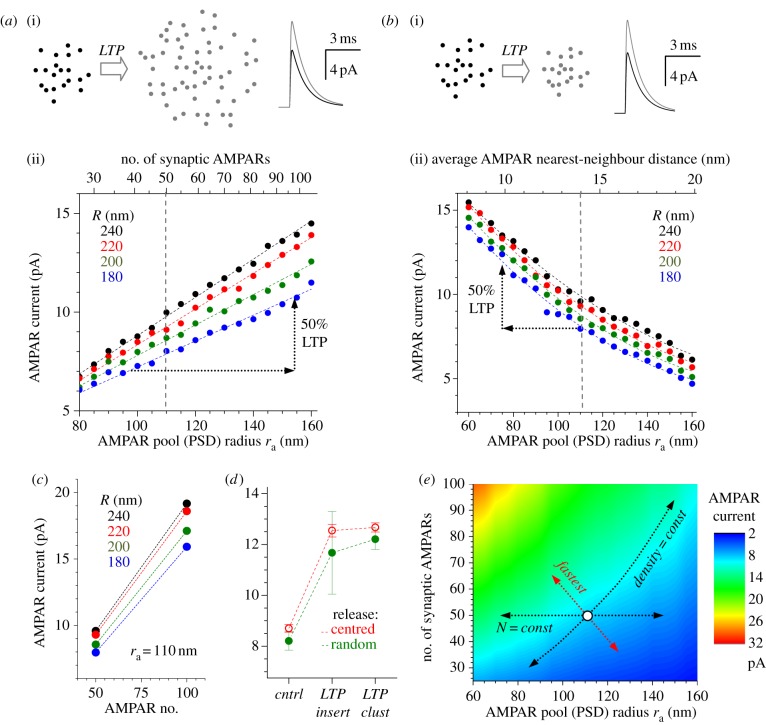
Potentiation of the synaptic AMPAR current can be achieved either by adding many more AMPARs to the pool, or by relatively modest clustering (crowding) of existing AMPARs, or both. (*a*) (i) Diagrams (not to scale) and traces: example simulated AMPAR currents before (black dots, trace) and after (grey) adding approximately 130% extra receptors to existing 50 AMPARs by expanding the receptor pool (PSD) from *r*_a_ = 110 nm to *r*_a_ = 160 nm (unchanged receptor density, synaptic cleft radius *R* = 200 nm). (ii) The relationship between the number of local synaptic AMPARs (or the PSD size) and the total AMPAR current under the unchanged surface density of AMPARs, for four synaptic cleft sizes *R*, as indicated; dots, results of individual simulation experiments; coloured dashed lines, linear regression; grey vertical dotted line (here and thereafter) indicates the experimental average size of the PSD at hippocampal CA3–CA1 synapses [26,33]; two dotted arrows illustrate an arbitrarily selected example of changes that corresponds to an approximately 50% potentiation of the AMPAR current. (*b*) (i) Diagrams (not to scale) and traces: example simulated AMPAR currents before (black trace) and after (grey) shrinking the pool of *N* = 50 AMPARs from *r*_a_ = 110 nm to *r*_a_ = 70 nm (PSD radius; *R* = 200 nm), with no changes in *N*. The relationship between the average inter-receptor nearest-neighbour distance (or the AMPAR pool size) and the total AMPAR current under the unchanged total number of AMPARs; coloured dashed lines, quadratic regression; other notations as in (*a*). (*c*) An example of the relationship between the synaptic cleft size (*R*), the total number of synaptic AMPARs and the total AMPAR current with the PSD (AMPAR pool) size remaining unchanged at *r*_a_ = 110 nm. (*d*) Testing the impact of glutamate release site variability. Red circles (mean±s.d., *n* = 20 trials), centre-fixed release site data (as in *a*–*c*) for the typical synapse in control conditions (*cntrl*, *r*_a_ = 110 nm, *R* = 200 nm, *N* = 50 AMPARs) and in conditions that correspond to approximately 50% AMPAR current potentiation, either through insertion of additional AMPAR to the periphery (*LTP insert*, *r*_a_ = 180 nm, *R* = 200 nm, *N* = 134 AMPARs) or by shrinking the PSD without any other changes (*LTP clust*, *r*_a_ = 75 nm). Green circles (mean ± s.d., *n* = 20 trials), data obtained under evenly random variation of the release site location over the active zone (opposite to the PSD); other synaptic and receptor parameters are the same as in control conditions (*cntrl*). (*e*) Parametric map depicting the relationship between the AMPAR pool size, receptor density and total AMPAR current (false colour scale). Circle, a reference configuration corresponding to the experimental average (*r*_a_ = 110 nm, *R* = 200 nm, *N* = 50 AMPARs); horizontal dotted arrows show how a change in the receptor density alone affects AMPAR current; curved dotted arrow shows how changes in the receptor numbers (unchanged density) affect the AMPAR current; red dotted arrows depict the direction of the fastest change.

We first asked how rapidly the synaptic AMPAR current increases with new AMPARs added to the PSD (with the PSD expanding in area as new AMPARs are added) assuming that the average surface density (‘packing’) of AMPARs remains unchanged. Simulation results indicated that, for a variety of synaptic cleft sizes (*R*), the relationship between the number of added AMPARs and the synaptic current is relatively weak: to increase the current by approximately 50%, the receptor numbers have to be boosted by 100–200% (figure 2*a*). This relationship was qualitatively similar for various cleft dimensions and across the range of AMPAR pool (PSD) sizes. The simple reason for such a disproportionally weak effect is that AMPARs added to the PSD periphery are much less sensitive to glutamate released near the PSD centre compared with AMPARs expressed closer to the release site. These simulations also indicated that, in principle, expanding the synaptic cleft (*R*) on its own could also boost the synaptic current, with the effect growing stronger at higher AMPAR densities. Again, this relationship is due to the simple fact that the two-dimensional cleft corresponds to greater (i.e. stronger and longer) local transients of released glutamate compared to the three-dimensional extracellular space surrounding it.

### (b) Modest crowding of synaptic AMPARs on the nanoscale can boost synaptic current without any new receptors

We next changed the scenario and asked whether the contraction of the synaptic AMPAR pool, i.e. a reduction in the average distance between receptors without any changes in the receptor numbers, would produce detectable changes in the total AMPAR current. Perhaps surprisingly, our data predicted that approximately 50% boost in the total current requires a relatively modest decrease in the inter-receptor distance (30–35%; figure 2*b*). Again, this effect remained robust for different synaptic cleft dimensions and over a wide range of AMPAR pool (PSD) sizes that reflect the average nearest-neighbour distances between individual AMPARs. Alternatively, inserting new receptors to the PSD without changing the PSD size, i.e. increasing both AMPAR number and their surface density, had a combined boosting effect on the AMPAR current (figure 2*c*).

### (c) Varying the intra-cleft location of glutamate release

Although in the above simulations the glutamate release site was placed at the cleft centre, this may not necessarily be the case in reality: CA3–CA1 synapses display a range of locations for docked (ready-releasable) synaptic vesicles within the presynaptic active zone [34]. Varying the release site location in a synapse model was previously demonstrated to reduce the average synaptic current while substantially increasing its amplitude variability [16]. To assess the effect of this variation on our conclusions, we explored the characteristic scenario in which the typical synapse (*r*_a_ = 110 nm, *R* = 200 nm, *N* = 50 AMPARs) underwent approximately 50% potentiation either by adding AMPARs at the same density or by increasing AMPAR density without changing their number. We therefore first looked at this scenario with the centre-fixed glutamate release site and then repeated simulations for exactly the same receptor arrangements and the same synaptic architecture but with the release site being randomly distributed, trial-to-trial, over the presynaptic active zone which opposes the PSD. The comparison showed that the stochastic occurrence of the release site generally decreased the AMPAR current amplitude while substantially increasing its variability (figure 2*d*), which was consistent with previous estimates [16]. At the same time, however, the effect of AMPAR pool expansion or clustering on the synaptic current potentiation (approx. 50% LTP) was generally indistinguishable from that observed in the centre-fixed release scenario (figure 2*d*).

### (d) A certain combination of modest changes in receptor density and numbers alters synaptic efficacy ‘most efficiently’

To evaluate and compare relative contributions of various changes in receptor arrangement (and synaptic configuration) leading to potentiation or depression of the AMPAR current, we explored the synaptic parameter space more systematically. The resulting parametric map (figure 2*e*) helps us to understand the pattern of synaptic current alterations pertinent to changes in the AMPAR pool (PSD) size or in the total AMPAR number (and thus the average inter-receptor distance), or in both parameters simultaneously. For instance, these data illustrate quantitatively to what degree the synaptic AMPAR current is sensitive to a variation in the AMPAR numbers at a constant surface density (figure 2*e*, curved dotted arrows marked *Density* = *const*), or to receptor clustering with no extra receptors (figure 2*e*, horizontal dotted arrows marked *N* = *const*). They also indicate that a certain type of synaptic receptor rearrangement can alter the synaptic response in a most ‘economical’ way, i.e. by implementing the smallest, in relative terms, structural alteration of the synapse. This type of change corresponds to the steepest gradient for each data point which reflects a given synaptic configuration (figure 2*e*; exemplified by the arrow marked *fastest*).

### (e) Synaptic responses are also sensitive to NMDAR clustering

**Figure 3.**
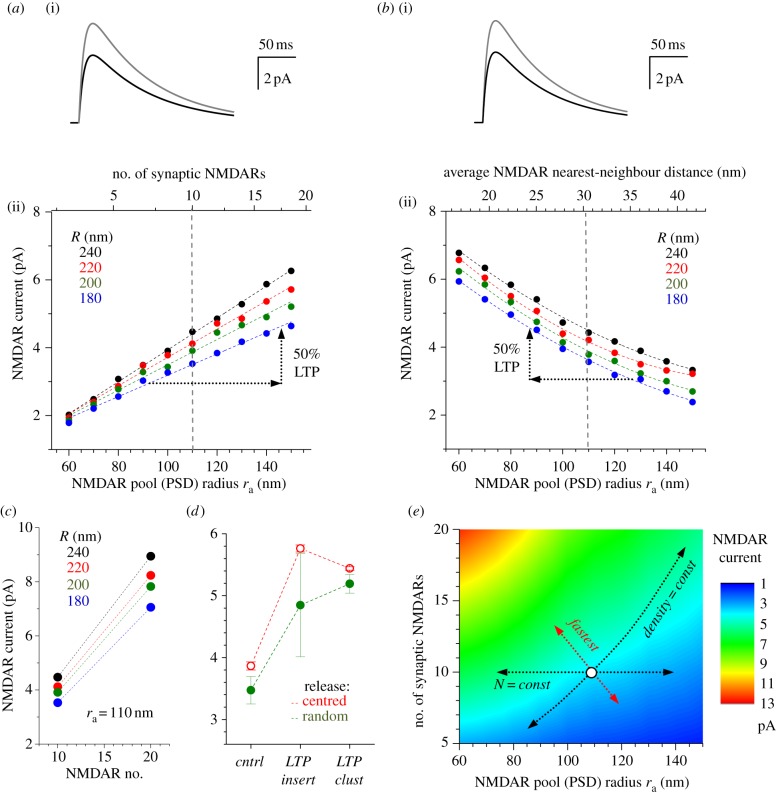
Potentiation of the synaptic NMDAR current can be achieved either by adding more NMDARs to the pool, or by clustering (crowding) of existing NMDARs, or both. (*a*) (i) Traces: example simulated NMDAR currents before (black) and after (grey) adding 12 new receptors (120% extra receptors) to existing 10 NMDARs by expanding the receptor pool from *r*_a_ = 110 nm to *r*_a_ = 165 nm (NMDAR density unchanged, *R* = 200 nm). (ii) The relationship between the number of local synaptic NMDARs (or the NMDAR pool size) and the total NMDAR current, with the unchanged NMDAR density. Two dotted arrows illustrate an arbitrarily selected example of changes that corresponds to approximately 50% potentiation of the NMDAR current. Other notations are as in figure 2*a*. (*b*) (i) Traces: example simulated NMDAR currents before (black) and after (grey) shrinking the pool of *N* = 10 NMDARs from *r*_a_ = 110 nm to *r*_a_ = 73 nm (*R* = 200 nm), with no changes in *N*. The relationship between the average inter-receptor nearest-neighbour distance and the total NMDAR current under the unchanged total number of NMDARs; coloured dashed lines, quadratic regression. Other notations are as in (*a*). (*c*) An example of the relationship between the synaptic cleft size (*R*), the total number of synaptic NMDARs and the total NMDAR current with the PSD (NMDAR pool) size remaining unchanged at *r*_a_ = 110 nm. (*d*) Testing the impact of glutamate release site variability. Red circles (mean±s.d., *n* = 20 trials), centre-fixed release site data (as in *a–c*) for the typical synapse in control conditions (*cntrl*, *r*_a_ = 110 nm, *R* = 200 nm, *N* = 10 NMDARs) and in conditions that correspond to approximately 50% NMDAR current potentiation, either through insertion of additional NMDARs to the periphery (*LTP insert*, *r*_a_ = 190 nm, *R* = 200 nm, *N* = 30 NMDARs) or by shrinking the PSD without any other changes (*LTP clust*, *r*_a_ = 80 nm). Green circles (mean±s.d., *n* = 20 trials), data obtained under evenly random variation of the release site location over the active zone (opposite to the PSD); other synaptic parameters are the same as in control conditions (*cntrl*). (*e*) Parametric map depicting the relationship between the NMDAR pool (PSD) size, NMDAR density and total NMDAR current (colour scale); circle, a reference configuration corresponding to the experimental average (*r*_a_ = 110 nm, *R* = 200 nm, *N* = 10 NMDARs); horizontal dotted arrows show how a change in the receptor density alone affects NMDAR current; curved dotted arrow shows how changes in the receptor numbers (unchanged density) affect the NMDAR current; red dotted arrows depict the direction of the fastest change.

The estimates above suggest that simply expanding the PSD by adding new AMPARs is a considerably less ‘efficient’ way to boost the synaptic strength when compared with increases in the AMPAR density. Because many types of excitatory synapses express another common ionotropic glutamate receptor, NMDAR, it was important to understand what changes in the NMDAR expression and pattern could potentiate the synaptic NMDAR current. We therefore carried out a theoretical exploration of NMDAR activation by glutamate in conditions similar to those we considered for AMPARs (figure 3). Although activation of higher affinity NMDARs is supposed to be much less sensitive to the distance from the glutamate release site compared with lower affinity AMPARs [18,35,36], our simulation results indicate that this distinction between AMPARs and NMDARs is less prominent inside the cleft. Indeed, the NMDAR current was also sensitive to both adding NMDARs to the PSD and to NMDAR clustering, although the difference between the two effects was somewhat less striking compared with that in the case of AMPARs (figure 3*a–c*). Similar to the case of AMPARs, varying the release site location reduced the average amplitude of NMDAR current while increasing its variability (figure 3*d*). At the same time, the effect pertinent to LTP expression remained qualitatively similar (figure 3*d*). Again, a systematic exploration of synaptic parameters provided intuitive clues as to what type of changes in the NMDAR expression or pattern could produce the fastest, and thus arguably the most ‘efficient’, alteration in the NMDAR current (figure 3*e*).

## 4. Discussion

Here, we have employed a Monte Carlo model of small central synapses to examine what type of changes in local AMPAR (and NMDAR) expression might explain the receptor current increase following the induction of LTP, assuming no changes in the amount of glutamate released. The results indicate that the addition of new AMPARs simply by expanding the PSD results in a disproportionally small boost of the AMPAR current. On average, a given fractional increase in the AMPAR current requires a three to four times larger increase in the relative number of AMPARs added this way. By contrast, a relatively modest (approx. 30%) reduction in the average inter-AMPAR distances within the PSD, and no additional AMPARs, could provide a robust (approx. 50%) rise in the AMPAR current amplitude. Our tests suggested that this conclusion was largely independent of whether the glutamate release site was fixed at the cleft centre or varied randomly over the active zone. This outcome was held over a wide range of poorly controlled synaptic parameters, suggesting that the conclusion was relatively robust. Importantly, we also found that for a given synaptic architecture, there is a certain combination of changes in AMPAR (and NMDAR) density and numbers that could provide the ‘most economical’, in terms of the relative structural change, path to the required level of synaptic potentiation or depression.

Notwithstanding the lack at present of direct experimental evidence supporting the hypothesis of use-dependent AMPAR nanoscale clustering, there are several factors, in addition to being by far the most parsimonious explanation, which make this hypothesis intellectually attractive. Firstly, this mechanism is readily consistent with the fact that LTP reflects an increased number of *activated* postsynaptic AMPARs. Because AMPARs are normally far from saturation at the synapses under study [22,37–39], the expression of LTP might, in principle, reflect either activation of newly inserted AMPARs or a higher activation level of the existing AMPARs, or perhaps both. Secondly, because AMPAR clustering does not require an increase in the total receptor numbers expressed within the PSD, the involvement of complex cellular machineries engaged in use-dependent AMPAR trafficking might not necessarily be critical throughout all stages of LTP expression. In this context, it is noteworthy that manifestation of AMPAR trafficking is usually detected minutes after the plasticity-inducing stimulus, whereas electrophysiological indicators point to a virtually immediate synaptic efficacy increase following LTP induction. Indeed, it is reasonable to think that structural rearrangement on the nanoscale is likely to occur more rapidly than changes associated with molecular transport over macroscopic dendritic compartments. Thirdly, the fact that some of the molecular cascades responsible for AMPAR trafficking have been found as a prerequisite for LTP expression as such might also reflect an important role of the same molecular machinery for nanoscale AMPAR clustering.

Our results suggest that activation of high-affinity NMDARs is also sensitive to the clustering inside the synaptic cleft. It is tempting to speculate that this mechanism could, in principle, contribute to activity-dependent changes in NMDAR-mediated postsynaptic Ca^2+^ entry. The latter in turn could affect local conditions for Ca^2+^-dependent plasticity. NMDARs are thought to undergo much slower macro- and microscopic movements compared with AMPARs, also depending on the receptor isoform [40,41]. Their ‘reluctance’ to move suggests that small nanoscale rearrangement of NMDARs could play a significant role in plasticity changes involving NMDAR signalling.

The features of synaptic receptor crowding on the nanoscale are presently beyond the resolution of optical microscopy or other real-time recording methods, making it difficult to test and explore the underlying machinery in a direct fashion. Nonetheless, the typical PSD of central synapses appears to have enough space to accommodate variable numbers of AMPARs and NMDARs at a range of surface densities [42]. This suggests that use-dependent changes in receptor crowding within the PSD are not implausible. Interestingly, the key PSD constituent, the PSD-95 protein, appears to give rise to scaffolding-like filamentous links that space synaptic AMPARs and NMDARs at relatively regular 20–30 nm intervals [43], which appear somewhat greater than the inter-receptor distances explored in our simulations. However, freeze-fracture replica labelling studies reveal smaller intervals between individual AMPARs in the PSD [44]. More importantly, our simulations quote the average nearest-neighbour distance assuming a uniformly random receptor distribution (Poisson point process), whereas this characteristic distance is substantially greater for a regular expression pattern, for instance a two-dimensional square lattice, with the same surface density [45]. The aim of our study was to explore several hypotheses (pertinent to the microscopic mechanisms of LTP expression) that would appear physiologically plausible and yet consistent with the existing experimental evidence. It would require, however, a new generation of experimental techniques to test what actually controls the receptor expression pattern inside the synaptic cleft and whether this pattern indeed changes in the course of use-dependent plasticity.
